# Determinants of Willingness to Share Wearable Health Data with Health Care Providers in Appalachian Populations: an Exploratory Study

**DOI:** 10.13023/jah.0701.04

**Published:** 2025-05-01

**Authors:** Gilbert Munoz Cornejo, Joonghee Lee

**Affiliations:** Appalachian State University; Appalachian State University

**Keywords:** Appalachia, data sharing, digital health behaviors, health status, healthcare providers, sociodemographic, wearable health devices

## Abstract

**Background:**

Wearable health devices capture metrics (e.g., physical activity, ECG, sleep) that can enhance care when shared with providers. Yet, willingness to share wearable data may differ in Appalachia, where chronic disease burdens, mistrust, and limited infrastructure pose unique challenges.

**Objective:**

This study explored (1) which sociodemographic, health, and digital behaviors correlate with willingness to share wearable data and (2) how these insights can guide region-specific interventions in Appalachia.

**Methods:**

We analyzed 320 Appalachian respondents from the Health Information National Trends Survey (HINTS 6). Descriptive statistics and logistic regression models examined willingness to share wearable data. Because of small cell counts, we supplemented with a Firth (penalized) logistic regression for robustness.

**Results:**

Approximately 25.0% unweighted (27.9% weighted) were willing to share wearable data, but two-thirds did not respond or were inapplicable. The final adjusted model (n=47) revealed:

- Income: Higher income correlated with increased willingness (e.g., aOR=8.52e+04 for $35–49k vs.
- Self-Rated Health: “Good” or “very good” health was associated with higher odds of sharing than “poor” health (aOR=4406.52; p<.05).
- Messaging: Surprisingly, participants who never messaged providers showed greater willingness (aOR=1.93e+07; p

**Conclusions:**

These preliminary findings suggest that household income, perceived health, and digital behaviors influence wearable data-sharing in Appalachia, whereas national demographic trends may not apply. Future work should use larger samples, mixed methods, and region-specific approaches to address mistrust, privacy concerns, and infrastructural barriers, aiming to enhance remote patient monitoring and reduce health disparities.

## INTRODUCTION

Wearable health device technologies have expanded rapidly in both clinical and consumer markets,^1^,^2^ offering the potential to transform health care delivery through continuous, real-time patient data.^3^–^6^ Currently, an estimated 21% of United States (U.S.) adults use wearable devices, highlighting their growing acceptance.^1^ These devices — ranging from physical activity trackers to electrocardiogram (ECG) sensors and smartwatches — capture key health metrics such as physical activity, heart rate, and sleep patterns that, when shared with healthcare providers, can enhance care quality, improve coordination, facilitate treatment development, and optimize health outcomes.^7^–^15^

Despite their potential, research underscores significant socioeconomic and health-related challenges shaping digital health engagement across diverse populations. For example, Caine and Hanania^16^ found that patients want specific control over the sharing of sensitive protected health information (PHI) with health care providers, while Grande et al.^17^ showed that willingness to share digital data for health-related uses is strongly influenced by both the type and source of the information. Taken together, these findings illustrate that health data-sharing concerns are highly context dependent, suggesting a need for tailored interventions to address both health and digital privacy barriers. Moreover, multiple obstacles continue to impede effective data sharing between patients and providers. Turner et al.^4^ identified both system-level issues (e.g., lack of reimbursement, poor interoperability with EHRs) and patient-level factors (e.g., privacy concerns, trust), and health care providers often struggle to interpret the volume and quality of patient-generated data.^4^ Notably, individuals who are younger, white, non-Hispanic, married, and have higher income and education levels are more likely to share health data.^4^ Nonetheless, willingness to share digital health information remains highly context dependent, notably shaped by socioeconomic factors^4^,^18^ and privacy concerns.^16^,^17^ Thus, the decision to share wearable data extends beyond technical considerations encompassing personal attitudes and broader socioeconomic contexts within the healthcare system.

Prior research has documented unique health and socioeconomic challenges in the Appalachian Region, characterized by disproportionately high rates of chronic obstructive pulmonary disease (COPD), infant mortality, cancer, cardiovascular disease, and substance misuse.^19^–^23^ Drug overdose, suicide, and alcoholic liver disease also occur at higher rates, contributing to a 37% increased mortality rate compared to non-Appalachian areas.^24^ These health burdens are further compounded by cultural mistrust of medical professionals,^25^ geographic isolation, limited transportation, underinvestment in healthcare, and technology constraints.^26^,^27^ Taken together, these factors can amplify or alter the common barriers to digital health engagement observed nationwide, underscoring the need for region-specific research.

Understanding the determinants of willingness to share wearable health data in Appalachian populations is especially critical for two reasons. First, it remains unclear whether national patterns — such as younger, more educated individuals being more inclined to share data — hold true in a region distinguished by unique health challenges and cultural dynamics. Second, Appalachian residents experience a disproportionately high burden of chronic disease and could benefit substantially from remote patient monitoring and digital health interventions. However, persistent barriers to data sharing — such as privacy concerns, mistrust, or limited healthcare infrastructure — risk undermining these potential gains. Despite these high stakes, research specifically examining how and why Appalachian residents choose to share (or not share) wearable data with healthcare providers is limited.

Accordingly, this study has two primary goals:

1. Identify sociodemographic, health, and digital usage characteristics associated with willingness to share wearable health data among Appalachian adults.
2. Generate exploratory insights that can inform tailored, region-specific strategies to improve remote patient monitoring and reduce long-standing health disparities.

By exploring the factors that shape data-sharing decisions in this underserved region, this study aims to establish an initial evidence base for designing culturally sensitive interventions, strengthening remote patient monitoring, and ultimately improving care outcomes in Appalachian communities.

## METHODS

### Sample and Data Source

HINTS is a probability-based, cross-sectional survey of the U.S. adult civilian, noninstitutionalized population that uses a two-stage, stratified sample design in which residential addresses are randomly selected, followed by the random selection of one adult respondent per household. The HINTS 6 response rate was 28.1% (N = 6,252), 320 of whom resided in Appalachian counties. Detailed descriptions of HINTS methodology are provided elsewhere.^28^ We included all Appalachian residents identified via the Appalachian Regional Commission (ARC) boundaries, which span thirteen states.^29^ Because this analysis used publicly available, deidentified data, institutional review board (IRB) approval was not required. Below we describe the principal measures used in the current analysis.

### Measures

#### Willingness to Share Wearable Health Data (Outcome)

Our primary outcome measure was the willingness to share data with health care providers. Participants who had used an electronic wearable device in the past 12 months were asked: “Would you be willing to share health data from your wearable device with your health care provider?” Response options included “Yes” (coded 1) or “No” (coded 2). Participants who were inapplicable (had not used a wearable; coded −1) or missing (−9, −7, etc.) were excluded from the final outcome analysis. Among respondents who answered (Yes/No), “Yes” indicates a willingness to share data with a provider. A more comprehensive description of all study variables, HINTS 6 items, and coding schemes is provided in Appendix A (Table 1A).

### Demographic, Health & Digital Usage Variables

We included the following demographic and health characteristics **(see Appendix A, Table 1A for details)**.

#### Demographic Variables

- Gender: Measured at birth, labeled Male (1) or Female (2). Missing or partial responses were coded −9 or −7.
- Race: Collapsed from multiple race categories into white, black, American Indian/Alaska Native (AI/AN), multiple, Asian/Pacific Islander, or missing. The final variable merges original multiple-race fields in our dataset.
- Education: Based on “What is the highest grade or level of schooling you completed?” (Education). For this analysis, categories were collapsed into: less than high school, high school graduate, some college, or college graduate.
- Marital Status: Participant responses (married, divorced, etc.) were recoded as “Currently Partnered” vs. “Not Currently Partnered,” plus a missing category.
- Household Income: Respondents were asked about total annual pre-tax income, then condensed categories into < $20,000, $20–34k, $35–49k, $50–74k, $75k+, plus a missing/no-response category.
- Age: A continuous measure from the question “What is your age?” Results reported both the unweighted mean ± SD and the replicate-weighted mean.
- Rural vs. Urban Classification: The survey used USDA Rural/Urban Continuum Codes (RUC 2003; variable name: RUC2003) to categorize county of residence. RUC2003 ranges from large metro areas (code 1) to completely rural, nonadjacent areas (code 9). For simplicity, the researchers collapsed codes 1–3 as Urban and 4–9 as Rural.

### Health & Digital Usage Variables

- General Health: A single-item measure, “In general, would you say your health is…?”, with response choices from 1 (Excellent) to 5 (Poor). Missing coded as −9, −7, or −1.
- Confidence in Managing Own Health: Coded 1 (Completely confident) to 5 (Not confident at all).
- Health Insurance: Determined by “Are you covered by any kind of health insurance…?” where 1=Yes, 2=No.
- Messaged Provider in Past 12 Months: “Send a message to a health care provider…?” (coded 1=Yes, 2=No).
- Shared Personal Health Info on social media: “In the past 12 months, how often did you share personal health info on social media?” We collapsed daily or weekly vs. monthly, never, or missing.

### Coding and Handling Missing Data

Responses coded as −9 (Not Ascertained), −7 (Partial), or −1 (Inapplicable) were treated as missing for analyses involving that variable. For certain items (e.g., wearable device usage), skip patterns led to inapplicability codes. These cases were removed prior to analyzing the outcome measure. Each variable’s final categories and missing codes are fully documented in Appendix A, Table 1A: Study Variables, HINTS 6 Items, and Coding Schemes.

### Data Analysis

We first generated both unweighted frequencies and percentages and survey-weighted estimates to describe demographics, health indicators, and digital usage characteristics; for continuous measures (e.g., age), unweighted means, standard deviations and replicate-weighted means provided raw and design-based perspectives, respectively. Data processing was performed in Jupyter Python Notebook using Pandas library and statistical analysis were carried out in R (version 4.3).

Each predictor was examined individually to evaluate its unadjusted association with the primary outcome (willingness to share wearable health data). Adjusted odds ratios (aORs) with 95% CIs were estimated to assess each variable’s contribution while controlling for other factors.^30^ A significance level of *p* < .05 was applied in all final tests. The multivariable logistic regression model thus constructed offers a comprehensive overview of how each retained predictor is related to the outcome when the effects of other factors are simultaneously considered.^30^

Model fit was assessed using Hosmer–Lemeshow tests, classification tables, AIC/BIC comparisons, and receiver operating characteristic (ROC) curves to evaluate discrimination (area under the curve, AUC). Missing values were handled by listwise deletion, given the modest sample size and exploratory nature of the study.^30^ Finally, to address potential small cell counts or quasi-separation, we conducted a supplementary penalized (Firth) logistic analysis as a robustness check; while useful for stabilizing parameter estimates in sparse data contexts, it does not accommodate replicate survey weights.^31^

## RESULTS

### Descriptive Characteristics

Table 1 presents demographic and health-related characteristics of the 320 Appalachian respondents. Unweighted frequencies indicated that 56.25% were female, whereas the survey-weighted proportions suggested 52.09% male. The unweighted mean age was 58.54 years (SD = 16.78), while the replicate-weighted estimate was 52.71 years. White participants comprised approximately three-quarters of the sample in both unweighted (75.00%) and weighted (75.15%) analyses. Urban residents represented 61.88% of the unweighted sample (68.77% weighted). Notably, 39.38% were college graduates unweighted, but the corresponding weighted proportion was 25.08%.

In total, 19.69% of participants reported having shared health information via a device or smartphone (18.23% weighted), and 45.00% (46.80% weighted) had messaged a provider in the past 12 months. The primary outcome — willingness to share wearable health data — was reported by 25.00% of respondents unweighted (27.91% weighted). However, over two-thirds (68.75% unweighted; 67.26% weighted) either did not respond or were inapplicable (survey skip patterns), reducing the analytic sample for regression models.

### Unadjusted (Bivariate) Logistic Regression

Table 2 (left columns) shows single-predictor logistic regressions using unweighted data. Being not currently partnered versus partnered was associated with lower odds of willingness (*OR* = 0.20, 95% CI [0.04, 0.95], *p* = .043). Participants identifying as Asian/Pacific Islander had significantly lower unadjusted odds of willingness relative to white counterparts (*OR* = 0.02, 95% CI [0.002, 0.18], *p* = .001). Several other factors such as education and income showed suggestive but non-significant relationships.

### Adjusted Multivariable Model

The final multivariable logistic regression model (Table 2, right columns) included all hypothesized predictors but retained only 47 participants due to listwise deletion for missing or skipped items. Many coefficient estimates were extremely large or small (e.g., female gender *OR* = 0.0000232, 95% CI [1.38e− 08, 0.039], *p* = .006), indicating quasi-separation and sparse data in some categories. Several variables still reached *p* < .05 such as higher income, “good” or “very good” health, being uninsured, not messaging a provider.

### Model Fit Diagnostics

We used Hosmer–Lemeshow tests, classification tables, AIC/BIC comparisons, and receiver operating characteristic (ROC) curves to evaluate the adjusted logistic model. Although no single criterion definitively signaled a good or bad fit, the small sample (n = 47) and pronounced quasi-separation hinder a robust assessment. To address these issues, a supplementary Firth (penalized) logistic regression was conducted using 79 observations that had fewer missing values (Appendix, Table 2A). Firth correction can help stabilize estimates in sparse data conditions but does not accommodate survey weights (30, 31). In this penalized model, no predictors achieved statistical significance, and the global Wald test was non-significant (*χ^2^*(29) = 9.88, *p* = 0.9996).

## IMPLICATIONS

This exploratory study examined the willingness of Appalachian residents to share wearable health data, an initial foray into a topic that remains underexplored in a region with longstanding healthcare disparities. Given the high degree of missingness, the small analytic sample, and signs of quasiseparation in our model, the findings and inferences should be served as preliminary. They serve primarily as hypothesis-generating, signaling areas that warrant more robust future research. Although one might expect that individuals with poorer health would be more inclined to share data due to heightened clinical need, this preliminary analysis indicates otherwise. Specifically, respondents reporting “good” or “very good” health demonstrated higher odds of willingness to share their data compared to those in “poor” health — a result that could reflect privacy concerns outweighing perceived need, in line with prior work.^16^

The data also suggest that higher household income may facilitate sharing, echoing research linking socioeconomic resources to technology adoption.^4^ In contrast, participants who had not messaged their providers or never shared personal health information on social media had high odds of willingness, a finding possibly reflecting contextual attitudes toward privacy, mistrust of formal healthcare channels,^16^,^25^ or simply statistical anomalies due to our sparse data.

It is important to underscore that demographic factors such as age, gender, and education did not emerge as clear predictors here once quasi-separation and missingness were accounted for echoing literature in the topic.^4^ Neither were there significant differences between rural and non-rural participants—a result suggesting that, in the Appalachian Region, barriers may extend beyond purely technological access (e.g., broadband) to include possibly healthcare experiences, or trust in digital tools.

A major strength of this study is its focus on a region — Appalachia — where little research has addressed wearable technology and data-sharing behaviors. Nonetheless, several limitations underscore its exploratory nature: First, over two-thirds of participants did not provide a response to the primary outcome, leading to a reduced analytic sample and introducing quasi-separation in certain predictor categories. Second, the willingness to share wearable data was assessed by a single self-reported question, potentially oversimplifying a nuanced decision-making process involving privacy, trust, and health beliefs.

Third, extremely large or small odds ratios in the adjusted model highlight data instability. A supplementary Firth regression attempted to mitigate separation but yielded no significant predictors, reflecting the complexity of these data (Appendix Table 2A). These findings provide compelling initial evidence that highlights key factors shaping wearable data-sharing in Appalachia and establishes a robust foundation for future research.

The implications for healthcare providers, policymakers, and researchers emerging from this exploratory study are comprehensive. Developing tailored interventions is critical, as individuals with higher self-rated health and those who have not previously engaged in health-information sharing may be more receptive to wearable-based health promotions. Therefore, providers could target pilot programs toward both well-managed patients and at-risk groups. Addressing data privacy and trust is equally important, with qualitative studies follow-ups needed to explore specific privacy concerns, cultural beliefs, and trust barriers that shape willingness to share health data in the region.^25^ Leveraging socioeconomic insights, policymakers should implement targeted financial support initiatives to enhance digital participation among lower-income populations. Research demonstrates that cost barriers significantly limit technology adoption and broadband access among disadvantaged groups.^2^,^18^ Finally, expanding community-based solutions through mixed-methods or longitudinal studies can investigate how Appalachian residents navigate cultural mistrust, geographic isolation, and limited digital infrastructure. Integrating wearable monitoring into telehealth^32^ services may offer a promising avenue to reach underserved populations and improve chronic-disease outcomes.

Building on the present exploratory findings, future research should (1) encompass larger, longitudinal designs to track real-world data-sharing behavior over time and assess whether shifts in policy or new technologies influence willingness; (2) incorporate mixed-methods approaches—such as indepth interviews or focus groups—to capture the nuanced dynamics of trust, cultural beliefs, and perceived benefits or drawbacks of sharing; and (3) evaluate intervention efficacy through pilot programs or randomized trials that introduce wearable devices with supportive education, identifying strategies that effectively bolster engagement and ultimately improve health outcomes in the Appalachian Region.

This study provides a crucial starting point for understanding willingness to share wearable health data in Appalachia. While high missingness and quasiseparation limit definitive interpretations, the potential influence of self-rated health, income, and past sharing behaviors offer direction for targeted interventions. As digital health technologies evolve, tailored approaches — grounded in local culture and trust-building strategies — could meaningfully expand wearable data usage to improve chronic disease management and reduce long-standing health disparities in Appalachian communities.

**SUMMARY BOX**

**What is already known about this topic?**

National research suggests that demographic factors (e.g., younger age, higher education) often correlate with increased willingness to share wearable health data. Appalachia faces disproportionately high burdens of healthcare issues and limited healthcare access, yet little is known about how these factors may influence individuals’ decisions to share wearable health data.

**What is added by this report?**

This exploratory study offers preliminary insights specific to Appalachian communities, where digital health engagement remains understudied despite well-documented health disparities. Within a small analytic sample, higher income and better self-rated health emerged as possible predictors of greater willingness to share wearable data. Additionally, individuals who had never messaged a provider or posted personal health information on social media also demonstrated high odds of willingness—potentially reflecting distinctive attitudes toward formal healthcare channels. However, substantial nonresponse (over two-thirds) and quasi-separation indicated that these findings should be treated as hypothesis-generating.

**What are the implications for future research?**

Larger-scale, mixed-methods, and longitudinal studies are needed to confirm these preliminary signals, refine our understanding of sociodemographic influences, and address cultural mistrust in Appalachian communities. Innovative interventions—such as targeted financial support for devices, culturally tailored education, and community-based pilot programs—could help expand wearable data sharing and reduce persistent health disparities in rural Appalachia.

## Supplementary Information



## Figures and Tables

**Table 1:** Descriptive Characteristics of Appalachian Sample (N=320), Unweighted and Weighted

Variable	Category	Unweighted n (%)	Weighted %
**Willingness to Share (Outcome)**	No	20 (6.25)	4.83
	Yes	80 (25.00)	27.91
	Missing	220 (68.75)	67.26
**Gender**	Male	127 (39.69)	52.09
	Female	180 (56.25)	42.21
	Missing	13 (4.06)	5.70
**Race**	White	240 (75.00)	75.15
	Black	47 (14.69)	8.09
	AI/AN	1 (0.31)	0.61
	Multiple	6 (1.88)	4.25
	Asian/PI	10 (3.12)	3.77
	Missing	16 (5.00)	8.14
**Education**	Less than HS	23 (7.19)	6.67
	High school grad	76 (23.75)	29.08
	Some college	83 (25.94)	33.62
	College grad	126 (39.38)	25.08
	Missing	12 (3.75)	5.55
**Marital Status**	Currently partnered	157 (49.06)	56.28
	Not currently partnered	150 (46.88)	38.10
	Missing	13 (4.06)	5.62
**Income**	< $20,000	62 (19.38)	13.23
	$20–34k	48 (15.00)	13.70
	$35–49k	48 (15.00)	11.96
	$50–74k	41 (12.81)	12.94
	$75k+	93 (29.06)	39.54
	Missing	28 (8.75)	8.63
**Age (years)**	Mean ± SD (unweighted)	58.54 ± 16.78	—
	Weighted Mean	—	52.71
**Rural/Urban**	Urban	198 (61.88)	68.77
	Rural	122 (38.12)	31.23
**General Health**	Excellent	31 (9.69)	12.28
	Very good	96 (30.00)	33.66
	Good	128 (40.00)	35.26
	Fair	47 (14.69)	11.39
	Poor	9 (2.81)	2.95
	Missing	9 (2.81)	4.46
**Confidence in Managing Own Health**	Completely confident	91 (28.44)	26.25
	Very confident	133 (41.56)	38.08
	Somewhat confident	68 (21.25)	24.27
	A little confident	15 (4.69)	5.79
	Not confident at all	5 (1.56)	1.09
	Missing	8 (2.50)	4.52
**Health Insurance**	Yes	298 (93.12)	91.78
	No	16 (5.00)	4.68
	Missing	6 (1.88)	3.54
**Messaged Provider in Past 12 Months**	Yes	144 (45.00)	46.80
	No	96 (30.00)	32.44
	Missing	80 (25.00)	20.75
**Shared Personal Health Info on Social Media**	Almost daily	4 (1.25)	1.04
	1/week+	1 (0.31)	0.14
	Few times/mo.	4 (1.25)	0.72
	Less than mo.	28 (8.75)	9.36
	Never	273 (85.31)	87.39
	Missing	10 (3.12)	1.36

NOTE:

Unweighted columns show raw frequencies (n) and row percentages; weighted columns reflect replicate-weighted percentages to account for the complex survey design. “Missing” includes participants who did not respond or were deemed inapplicable due to survey skip patterns (e.g., no wearable device). Age is presented as mean ± standard deviation (unweighted) and replicate-weighted mean.

**Table 2:** Unadjusted and Adjusted Multivariable Logistic Regressions Predicting Willingness to Share Wearable Data

Category	Predictor	Unadjusted OR (95% CI)	*p*	Adjusted OR (95% CI)	*p*
**Age** (Continuous)	Age	1.04 (0.98, 1.09)	.173	1.30 (0.99, 1.71)	.058
**Gender** (Ref: Male)	Female	1.10 (0.25, 4.93)	.899	0.0000232 (1.38e−08, .039)	.006^**^
**Race** (Ref: White)	Black	0.73 (0.10, 5.41)	.759	2.52 (0.018, 360.25)	.710
	AI/AN only	(empty)	—	— (omitted)	—
	Multiple	1.05 (0.09, 12.35)	.970	0.0000465 (8.16e−10, 2.65)	.073
	Asian/PI	0.02^**^ (0.002, 0.18)	.001	4.49e−09 (2.01e−13, .00010)	.000^**^
**Education** (Ref: <HS)	High School	1.64 (0.09, 28.98)	.734	5.32e−11 (2.41e−16,1.18e−05)	.000^**^
	Some College	5.80 (0.38, 88.98)	.204	5.11e−08 (7.46e−12,.00035)	.000^**^
	College Grad	1.94 (0.14, 26.30)	.617	4.03e−09 (4.36e−13,.000037)	.000^**^
**Marital Status** (Ref: Partnered)	Not Currently Partnered	0.20^*^ (0.04, 0.95)	.043	4.77 (0.0011,2.10e+04)	.710
**Income** (Ref: <$20k)	$20–34k	5.06 (0.54, 47.74)	.155	1.10e+06 (0.0596,2.02e+13)	.101
	$35–49k	1.35 (0.14, 12.57)	.793	8.52e+04 (1.89,3.84e+09)	.038^*^
	$50–74k	4.16 (0.48, 35.79)	.192	0.73 (0.0054, 97.00)	.896
	$75k+	3.45 (0.52, 22.84)	.196	1.79e+12 (1.26e+05,2.54e+19)	.001^**^
**Rural/Urban** (Ref: Urban)	Rural	0.64 (0.16, 2.54)	.517	0.0080 (3.84e−06,16.46)	.209
**Gen. Health** (Ref: Poor)	Very good	0.90 (0.12, 6.58)	.919	4406.52 (1.20,1.62e+07)	.045^*^
	Good	3.96 (0.47, 33.56)	.204	4.95e+10 (7.28e+04,3.36e+16)	.001^**^
	Fair	2.90 (0.26, 31.94)	.381	0.25 (1.44e−05,4285.89)	.775
**Confidence in Managing Own Health** (Ref: Not)	Very conf.	0.25 (0.05, 1.22)	.086	0.94 (0.00059,1512.24)	.987
	Somewhat	1.46 (0.23, 9.28)	.686	5.37 (0.0057,5072.19)	.624
	A little	0.13 (0.01, 1.66)	.115	6.91e+11 (0.0065,7.38e+25)	.096
**Health Insurance** (Ref: Insured)	Uninsured	0.43 (0.05, 3.94)	.448	6.37e−09 (1.43e−14,0.0028)	.005^**^
**Messaged Provider in Past 12 Months** (Ref: Yes)	No Message	0.55 (0.11, 2.76)	.466	1.93e+07 (249.85,1.50e+12)	.004^**^
**Shared Personal Health Info on Social Media** (Ref: Weekly)	Never Shared Pers. Info	5.41 (0.40, 73.24)	.201	8.12e+11 (4.18,1.58e+23)	.039^*^

NOTES:

*p* < .05 = ^*^, ^*^p < .01 = ^**^. OR = Odds Ratio; CI = 95% Confidence Interval. Bivariate columns: single-predictor (unadjusted) logistic with unweighted n. Adjusted columns: final multivariable model (N=47) including all listed predictors. (empty) or (omitted) indicate perfect separation or no valid observations.
