# Supplementary material for: Determinants of Willingness to Share Wearable Health Data with Health Care Providers in Appalachian Populations: an Exploratory Study
Source: J Appalach Health. 2025 May 1;7(1):63–80. doi: 10.13023/jah.0701.04 (PMC12112009; doi:10.13023/jah.0701.04)
Supplement: Supplementary file 1 [file 7.1.4_Cornejo_Additionalfile.docx]

# Appendix

**Table 1A. Study Variables, HINTS 6 Items, and Coding Schemes**

| **Study Variables** | **Survey Question Text (Number)** | **HINTS Variable** | **Value Labels** | **Description / Notes** |
| --- | --- | --- | --- | --- |
| **Willingness to Share Wearable Health Data**(Outcome) | “B10a. Would you be willing to share health data from your wearable device with your health care provider?” | WillingShareData_HCP | 1 = Yes  2 = No  -1 = Inapplicable (no wearable device)  -9 = Missing (Not Ascertained),  -7 = Web partial, etc. | Only asked if WearableDevTrackHealth=1.  If participant had no wearable, coded -1. “Yes” indicates willingness to share data with provider. Missing → “.” |
| **Gender** | “R2. On your original birth certificate, were you listed as male or female?” | BirthGender | 1 = Male  2 = Female  -9 = Missing,  -7 = Web partial,  -5 = Multiple resp. error | Asked of all participants. Typically recoded to “Male” vs. “Female,” missing set to “.”. |
| **Race** | “R9. What is your race?” | Race_cat2_combined(custom) | White,  Black,  AI/AN,  Multiple,  Asian/PI,  Missing (collapsed from original multiple-race fields) | For analysis, race categories are combined into e.g., White, Black, AI/AN, Multiple, Asian/PI. Missing codes -9, -7 → “.” |
| **Education** | “R7. Highest grade or level of schooling completed?” | Education | 1=Less than 8 yrs  2=8–11 yrs  3=12 yrs or HS  4=Post-HS training  5=Some college  6=College grad  7=Postgrad | Often collapsed: <HS, HS, Some college, College grad. Missing code -9 or -7 → “.”. |
| **Marital Status** | “R6. What is your marital status?” | Maritalstatus_new_combine(custom) | Possibly 1=Currently Partnered (Married/Living as married)  2=Not Partnered (Divorced, Widowed, Separated, Single)  Missing if -9, -7 | Original categories recoded into Partnered vs. Not Partnered (+ missing). |
| **Household Income** | “R14. Combined annual pre-tax income.” | IncomeRanges | 1=$0–9,999  2=$10k–14,999  3=$15k–19,999 … up to 9=$200k+ | Collapsed: <$20k, $20–34k, $35–49k, $50–74k, $75k+. Missing code → “.”. |
| **Age** | “R1. What is your age?” | Age or Age Years | Numeric years. Missing = -9, -7, etc. | Self-reported. Typically presented as mean ± SD (unweighted) plus replicate-weighted mean. |
| **Geography: Rural vs. Urban** | USDA Rural/Urban Designation (2003). (No direct question; assigned by county) | RUC2003 (e.g., ruc2003_new_urb_rur) | 1=Metro≥1M,  2=Metro 250K–1M, 3=Metro<250K,  4–9=Nonmetro (various adjacency & population sizes).  Collapsed (1–3=Urban, 4–9=Rural). | Used to classify participants’ county of residence. No direct survey question. “Urban vs. Rural” is a typical binary recode. |
| **General Health** | “In general, would you say your health is…?” (H1) | GeneralHealth | 1=Excellent,  2=Very good,  3=Good,  4=Fair,  5=Poor,  Missing=-9, -7. | Single-item measure of self-rated health. Missing set to “.”. |
| **Confidence in Managing Own Health** | “H2. Overall, how confident are you about your ability to take good care of your health?” | OwnAbilityTakeCareHealth | 1=Completely confident… 5=Not confident at all,  Missing=-9, -7. | Higher numeric values = lower confidence, if used as-is. Often reversed or recoded. |
| **Health Insurance** | “C6. Are you covered by any kind of health insurance…?” | HealthInsurance2 | 1=Yes,  2=No,  Missing=-9, -7 | If 2=uninsured. Missing set to “.”. |
| **Messaged Provider in Past 12 Months** | “B3b. In the past 12 months, have you used the Internet to send a message to a health care provider…?” | Electronic2_MessageDoc | 1=Yes,  2=no,  Missing=-9, -7. | Reflects electronic communication with provider. |
| **Shared Personal Health Info on Social Media** | “B12b. In the past 12 months, how often did you share personal health info on social media?” | SocMed_SharedPers | 1=Almost daily, 2=≥1/week,  3=A few times/mo., 4=<1/mo.,  5=Never,  Missing=-9, -7. | Frequency measure; sometimes simplified (Ever vs. Never). Missing recoded to “.”. |

**Table 2B: Firth Logistic Regression Predicting Willingness to Share Wearable Data (N = 79)**

| **Category** | **Predictor** | **B** | **SE** | **z** | **p** | **95% CI** |
| --- | --- | --- | --- | --- | --- | --- |
| **Age** (Continuous) | Age | 0.0030 | 0.03015 | 0.10 | 0.920 | -0.0561 , 0.0621 |
| **Gender** (Ref: Male) | Female | -0.6510 | 0.95590 | -0.68 | 0.496 | -2.5246 , 1.2225 |
| **Race** (Ref: White) | Black | -1.1158 | 2.02941 | -0.55 | 0.582 | -5.0933 , 2.8618 |
|  | AI/AN only | -2.1732 | 3.77348 | -0.58 | 0.565 | -9.5691 , 5.2227 |
|  | Multiple | -1.8904 | 1.71150 | -1.10 | 0.269 | -5.2449 , 1.4640 |
|  | Asian/PI | -1.7329 | 1.55871 | -1.11 | 0.266 | -4.7879 , 1.3221 |
| **Education** (Ref: <HS) | High school | -1.5958 | 2.19055 | -0.73 | 0.466 | -5.8892 , 2.6976 |
|  | Some college | -1.2653 | 2.19092 | -0.58 | 0.564 | -5.5594 , 3.0288 |
|  | College grad | -1.2067 | 2.00613 | -0.60 | 0.548 | -5.1386 , 2.7253 |
| **Marital Status** (Ref: Partnered) | Not Currently Partnered | 0.3259 | 1.01352 | 0.32 | 0.748 | -1.6605 , 2.3124 ) |
| **Income** (Ref: <$20k) | $20–34k | 0.0388 | 1.37557 | 0.03 | 0.978 | -2.6573 , 2.7348 |
|  | $35–49k | -0.3694 | 1.41688 | -0.26 | 0.794 | -3.1464 , 2.4076 |
|  | $50–74k | -0.3078 | 1.51497 | -0.20 | 0.839 | -3.2770 , 2.6615 |
|  | $75k+ | 1.9478 | 1.62709 | 1.20 | 0.231 | -1.2412 , 5.1369 |
| **Rural/Urban** (Ref: Urban) | Rural | -1.0190 | 0.83683 | -1.22 | 0.223 | -2.6591 , 0.6212 |
| **Gen. Health** (Ref: Poor) | Very good | 0.1656 | 1.43720 | 0.12 | 0.908 | -2.6513 , 2.9825 |
|  | Good | 1.5215 | 1.76144 | 0.86 | 0.388 | -1.9308 , 4.9739 |
|  | Fair | 1.3332 | 2.62087 | 0.51 | 0.611 | -3.8036 , 6.4700 |
| **Confidence in Managing Own Health** (Ref: Not confident at all) | Very confident | -0.7671 | 1.19726 | -0.64 | 0.522 | -3.1137 , 1.5795 |
|  | Somewhat | -2.1327 | 2.29510 | -0.93 | 0.353 | -6.6310 , 2.3656 |
|  | A little | -0.8455 | 2.55259 | -0.33 | 0.740 | -5.8485 , 4.1575 |
| **Health Insurance** (Ref: Insured) | Uninsured | -0.5573 | 1.37835 | -0.40 | 0.686 | -3.2588 , 2.1442 |
| **Messaged Provider in Past 12 Months** (Ref: Yes) | No | -2.2225 | 1.38936 | -1.60 | 0.110 | -4.9456 , 0.5006 |
| **Shared Personal Health Info on Social Media** (Ref: Weekly) | A few times/month | 0.5279 | 2.55488 | 0.21 | 0.836 | -4.4796 , 5.5354 |
|  | Less than once/month | 2.9468 | 2.26483 | 1.30 | 0.193 | -1.4922 , 7.3858 |
|  | Never | 1.0131 | 1.65014 | 0.61 | 0.539 | -2.2211 , 4.2473 |
|  |  |  |  |  |  |  |

**Notes:** Firth logistic is a penalized likelihood method used to handle separation. Results are unweighted (no survey weights). Categories with “(empty)” or “(omitted)” reflect perfect separation or collinearity. B = log-odds coefficient, SE = standard error, *z* = test statistic, *p* = p-value, 95% CI is the confidence interval for the log-odds scale. Overall model: *N = 79*, Wald χ²(29) = 9.88, p = 0.9996.
